# Effectiveness of a monovalent rotavirus vaccine in infants in Malawi after programmatic roll-out: an observational and case-control study

**DOI:** 10.1016/S1473-3099(14)71060-6

**Published:** 2015-04

**Authors:** Naor Bar-Zeev, Lester Kapanda, Jacqueline E Tate, Khuzwayo C Jere, Miren Iturriza-Gomara, Osamu Nakagomi, Charles Mwansambo, Anthony Costello, Umesh D Parashar, Robert S Heyderman, Neil French, Nigel A Cunliffe, for the VacSurv Consortium

**Affiliations:** a Malawi-Liverpool-Wellcome Trust Clinical Research Programme, College of Medicine, University of Malawi, Blantyre, Malawi; b Institute of Infection and Global Health, University of Liverpool, Liverpool, UK; c Epidemiology Branch, Division of Viral Diseases, National Center for Immunization and Respiratory Diseases, Centers for Disease Control & Prevention, Atlanta, GA, USA; d Graduate School of Biomedical Sciences, Nagasaki University, Nagasaki, Japan; e Ministry of Health & Population, Lilongwe, Malawi; f Institute of Global Health, University College London, London, UK; g Liverpool School of Tropical Medicine, Liverpool, UK

## Abstract

**Background:**

Rotavirus is the main cause of severe acute gastroenteritis in children in Africa. Monovalent human rotavirus vaccine (RV1) was added into Malawi's infant immunisation schedule on Oct 29, 2012. We aimed to assess the impact and effectiveness of RV1 on rotavirus gastroenteritis in the 2 years after introduction.

**Methods:**

From Jan 1, 2012, to June 30, 2014, we recruited children younger than 5 years who were admitted into Queen Elizabeth Central Hospital, Blantyre, Malawi, with acute gastroenteritis. We assessed stool samples from these children for presence of rotavirus with use of ELISA and we genotyped rotaviruses with use of RT-PCR. We compared rotavirus detection rates in stool samples and incidence of hospital admittance for rotavirus in children from Jan 1 to June 30, in the year before vaccination (2012) with the same months in the 2 years after vaccination was introduced (2013 and 2014). In the case-control portion of our study, we recruited eligible rotavirus-positive children from the surveillance platform and calculated vaccine effectiveness (one minus the odds ratio of vaccination) by comparing infants with rotavirus gastroenteritis with infants who tested negative for rotavirus, and with community age-matched and neighbourhood-matched controls.

**Findings:**

We enrolled 1431 children, from whom we obtained 1417 stool samples (99%). We detected rotavirus in 79 of 157 infants (50%) before the vaccine, compared with 57 of 219 (40%) and 52 of 170 (31%) in successive calendar years after vaccine introduction (p=0·0002). In the first half of 2012, incidence of rotavirus hospital admission was 269 per 100 000 infants compared with 284 in the same months of 2013 (rise of 5·8%, 95% CI −23·1 to 45·4; p=0·73) and 153 in these months in 2014 (a reduction from the prevaccine period of 43·2%, 18·0–60·7; p=0·003). We recruited 118 vaccine-eligible rotavirus cases (median age 8·9 months; IQR 6·6–11·1), 317 rotavirus-test-negative controls (9·4 months; 6·9–11·9), and 380 community controls (8·8 months; 6·5–11·1). Vaccine effectiveness for two doses of RV1 in rotavirus-negative individuals was 64% (95% CI 24–83) and community controls was 63% (23–83). The point estimate of effectiveness was higher against genotype G1 than against G2 and G12.

**Interpretation:**

Routine use of RV1 reduced hospital admissions for several genotypes of rotavirus in children younger than 5 years, especially in infants younger than 1 year. Our data support introduction of rotavirus vaccination at the WHO recommended schedule, with continuing surveillance in high-mortality countries.

**Funding:**

Wellcome Trust, GlaxoSmithKline Biologicals.

## Introduction

Rotavirus is the leading cause of severe acute gastroenteritis in infants and young children worldwide, causing about 453 000 child deaths every year before the introduction of the rotavirus vaccine.^1^ Widespread use of two orally administered, live attenuated rotavirus vaccines (a monovalent human rotavirus vaccine [RV1] and a pentavalent human–bovine reassortant rotavirus vaccine [RV5]) in North, Central, and South America, Europe, and Australia has largely reduced hospital admissions for rotavirus gastroenteritis, and decreased child deaths from diarrhoea in Mexico, Brazil, and Panama.^2^, ^3^, ^4^, ^5^, ^6^, ^7^, ^8^, ^9^

The greatest rotavirus burden, especially mortality, is in low-income countries in Africa and Asia. Clinical trials of RV1 and RV5 in Africa and Asia report lowest efficacy in the countries with the highest disease burden and lowest income.^10^, ^11^, ^12^ However, because of the high mortality from rotavirus gastroenteritis in such countries, in 2009 WHO recommended that all children should receive rotavirus vaccine, with a strong recommendation for countries where diarrhoeal diseases cause more than 10% of deaths.^13^

From January, 2012, to July, 2014, rotavirus vaccine was introduced in 19 countries in Africa.^14^ So far, no effectiveness data have been published from low-income countries in sub-Saharan Africa. Malawi is a very-low-income southern African country with a mortality in children younger than 5 years of 71 per 1000 livebirths^15^ and gross domestic product per person (purchasing-power parity) of US$900.^16^ With support from Gavi, the Vaccine Alliance, RV1 was introduced into Malawi's Expanded Programme on Immunisation on Oct 29, 2012. Two oral doses were scheduled to be given at 6 weeks and 10 weeks of age, without a catch-up campaign for older children. Building on previous studies of rotavirus gastroenteritis in Malawi, including a pivotal randomised placebo-controlled trial of RV1,^10^ we report observational data for the impact of a completed series of rotavirus vaccine against laboratory confirmed incidence of rotavirus diarrhoea hospitalisation in Malawi. We also report results of a case-control study that aimed to establish vaccine effectiveness with use of rotavirus-negative infants and community controls.

## Methods

### Study design

We did a hospital-based surveillance study of rotavirus disease in children younger than 5 years at one hospital in Malawi. We then did a case-control study to establish vaccine effectiveness against rotavirus gastroenteritis by assessing vaccine status of patients with gastroenteritis cases who tested positive for rotavirus with those who tested negative and with community controls. Our primary endpoint was the effectiveness of a completed series of rotavirus vaccine against laboratory confirmed rotavirus diarrhoea during routine vaccine use. Secondary endpoints were the genotype-specific effectiveness and the population impact of the RV1 vaccination programme.

### Surveillance and genotyping

From Jan 1, 2012, to June 30, 2014, we did active surveillance for acute gastroenteritis at Queen Elizabeth Central Hospital (QECH), Blantyre, Malawi, which is the referral hospital for the southern region of Malawi. QECH is the only inpatient referral facility that provides free health care to Blantyre district's 1 million residents. We enrolled children younger than 5 years who lived in Blantyre district and who presented at any time to the paediatric emergency department with acute gastroenteritis. Inclusion and exclusion criteria are contained in the appendix.

With written parental consent, we obtained demographic, clinical, and anthropometric data with use of a standardised case report form (for study definitions see appendix). We measured gastroenteritis disease severity with a modified Vesikari score.^17^ HIV status was established in finger-prick blood samples with two sequential antibody rapid tests (Determine, Abbott Laboratories, Germany, and Uni-Gold, Trinity Biotech, Ireland) or by DNA PCR in infants younger than 12 months, according to national guidelines.^18^ One stool sample was obtained for each child before discharge from the emergency department or within 48 h of hospital admission. We examined 10% faecal suspensions in phosphate buffered saline for rotavirus with ELISA (Rotaclone, Meridian Bioscience, Cincinnati, OH, USA). Rotavirus VP7 (G) and VP4 (P) genotypes were established for ELISA-rotavirus-positive specimens with qualitative, heminested multiplex reverse transcription PCR onsite at the hospital campus.^19^

We compared rotavirus detection rate and hospital admission incidence for rotavirus for Jan 1 to June 30 in the year before vaccine introduction (2012) with those for the same period in the first and second calendar year (Jan 1 to June 30, 2013 and 2014, respectively) after introduction. We calculated incidence of rotavirus hospital admissions as number of inpatient cases per 100 000 Blantyre mid-year population under surveillance; estimated with age-specific population projections from the 2008 population census.^20^ We derived projections for population through linear extension of the annual increase in age-specific population in the intercensal period from 1998 to 2008.

### Case-control study

In the same period, we also did a case-control study to establish vaccine effectiveness. From Oct 29, 2012, we recruited children with rotavirus gastroenteritis from the surveillance platform who fulfilled inclusion criteria (appendix) and who were vaccine age-eligible (6 weeks of age or older; born on or after Sept 17, 2012; thus were less than 6 weeks of age at introduction of vaccine). We recruited two control groups: unmatched vaccine age-eligible infants attending QECH with acute gastroenteritis who were negative for rotavirus with ELISA, and diarrhoea-free control individuals from the community whom we chose through a random walk method and who were matched to cases by date of birth (30 days older or younger for infants younger than 1 year, and 3 months older or younger if the child was older than 1 year) and subdistrict ward of residence. We obtained vaccine status of cases and controls from the patient held medical record (health passport) with capture of a digital image. We excluded from analysis children whose parents reported vaccine status but did not have a written record. We measured the odds of being vaccinated in cases and respective controls, and adjusted for age at admission and month and year of birth in unmatched test-negative controls; no adjustment was made in the matched analysis. Vaccine effectiveness was calculated as one minus the odds ratio derived from logistic regression, which was conditional in the matched study but not otherwise.

### Statistical analysis

We calculated that to assess the primary outcome using a case-control design, we needed 102 cases for 80% power to detect a vaccine effectiveness of at least 50% at two-sided 5% significance level, assuming vaccine coverage of 70%, a control to case ratio of four, and intracluster correlation coefficient within the matched groupings of 0·2.^21^ We did not do power calculations for secondary outcomes.

To calculate vaccine effectiveness, we subtracted the incidence rate ratio for rotavirus hospital admission for Jan 1 to June 30 in the year before vaccine introduction (2012) from one and compared this figure with the rate ratio for the same calendar months in the years after introduction (2013 and 2014).^22^ Additionally, to quantify the independent contribution of the vaccine programme to incidence over time, we used Poisson regression of rotavirus hospital admission incidence against vaccine coverage, adjusted for month of admission and stratified by age group. We extrapolated vaccine coverage for infants in Blantyre from the coverage in the rotavirus-test-negative infant cohort that we recruited. Vaccination coverage in children younger than 5 years assumed that children in the community not age eligible for vaccination were not vaccinated.

We tested differences in continuous covariates by *t* test or by Wilcoxon rank-sum test if not normally distributed based on normal quantile plots, and we examined categorical covariates with χ^2^ test. We did trend analysis for proportions with Royston's test.^23^ We did analyses with Stata 12·1.

Ethical approval was provided by the National Health Sciences Research Committee, Lilongwe, Malawi (867) and by the Research Ethics Committee of the University of Liverpool, Liverpool, UK (000490).

### Role of the funding source

The funders had no role in study design, collection, analysis and interpretation of data, writing of the report, or the decision to submit the paper for publication. We provided GlaxoSmithKline Biologicals SA with the opportunity to review a preliminary version of this manuscript for factual accuracy, but we were solely responsible for final content and interpretation. The corresponding author had full access to all the data from the study and had final responsibility for the decision to submit for publication.

## Results

We enrolled 1431 children younger than 5 years (1018 infants <12 months of age) with diarrhoea, from whom we collected 1417 stool specimens (99%). In this cohort of children, 1188 (82%) were being breastfed, 257 (18%) had been exposed to HIV, and 79 (6%) were infected with HIV (appendix). Among the specimens collected, most (872 [61%]) were from infants, and the most (1334 [94%]) were from children younger than 2 years.

**Figure 1:**
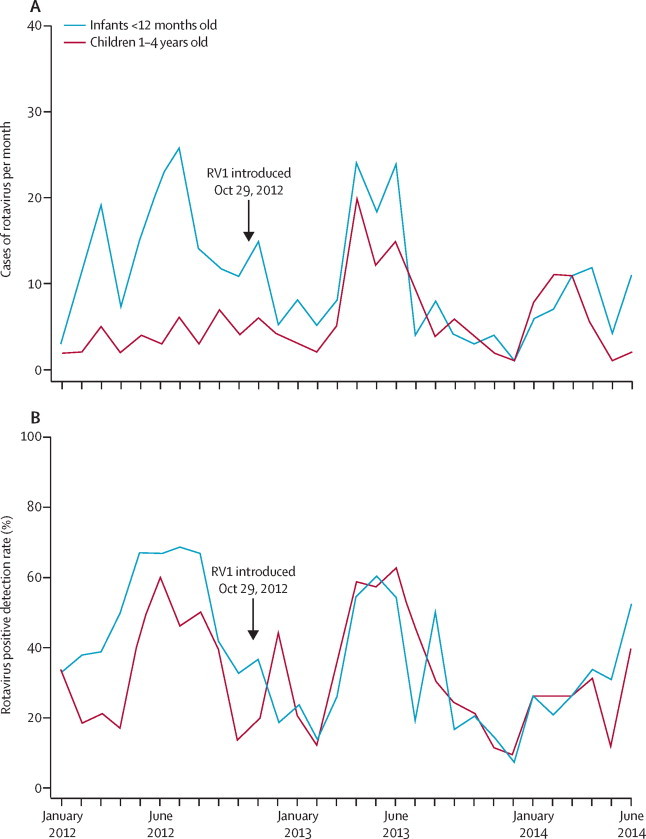
Rotavirus gastroenteritis detection by month in Queen Elizabeth Central Hospital, Blantyre (A) Number of rotavirus cases per month. (B) Proportion of stool samples positive for rotavirus.

**Figure 2:**
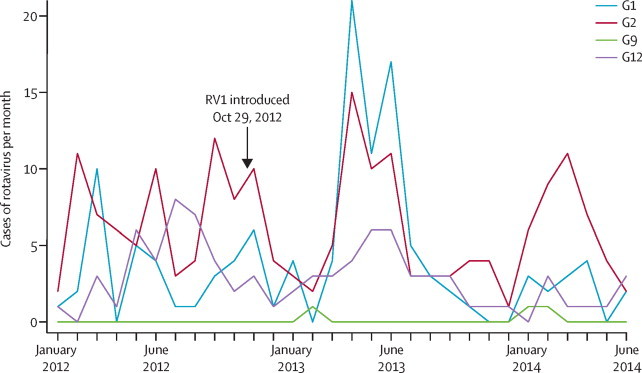
Rotavirus VP7 (G) genotypes detected by month in children younger than 5 years of age presenting to Queen Elizabeth Central Hospital, Blantyre, from Jan 1, 2012, to June 30, 2014 Graph excludes mixed infection.

Before vaccine introduction (Jan 1, 2012, to Oct 28, 2012), we collected 419 stool samples, of which 185 (44%) were positive for rotavirus (figure 1). After vaccine introduction (Oct 29, 2012, to June 30, 2014), we collected 998 stool samples, of which 318 (32%) were rotavirus positive (figure 1, appendix). Of 472 rotaviruses we examined from Jan 1, 2012, to June 30, 2014, prevalent genotypes included G2P[4] (117; 25%), G1P[8] (101; 21%), G12P[6] (48; 10%), and G2P[6] (47; 10%). 122 (26%) rotaviruses contained mixed G or P types or both (appendix). Genotype G1 was most common in the first year after the introduction of the vaccine, whereas genotype G2 dominated in the second year (figure 2).

The incidence analysis from January to June, 2012, 2013, and 2014, included 66% of all rotavirus-confirmed cases in infants and 66% of cases in children aged 1–4 years. From Jan 1, 2012, to June 30, 2012 (before introduction), rotavirus was detected in 98 of 225 (44%) children younger than 5 years, compared with 144 of 344 (42%; p=0·69) and 91 of 315 (29%; p<0·0001) children in the same months of 2013 and 2014, respectively (p for trend =0·0002). In the first 6 months of 2012, we detected rotavirus in 79 of 157 infants (50%; younger than 1 year) with gastroenteritis, compared with 87 of 220 (40%; p=0·04) in the same period of 2013, and 52 of 169 (31%; p<0·0001) in the same period of 2014 (p for trend=0·0002). The median age of patients with rotavirus gastroenteritis (cases) before vaccine introduction was 9·6 months (IQR 7·0–13·5), compared with 11·1 months (8·1–15·4) in 2014 (p=0·0001; appendix).

In the first 6 months of 2013, vaccine coverage in children younger than 5 years in Blantyre was 5%, rising to 18% in the first 6 months of 2014. From Jan 1 to June 30, 2012 (before vaccine introduction), the incidence of rotavirus-associated hospital admission per 100 000 children younger than 5 years was 120, compared with 178 during the same period in 2013 and 101 during the same period in 2014. In 2013, rotavirus hospital admissions were 49% higher (95% CI 13·6–94·2, p=0·004) than in 2012, but were 15% (−14·7 to 37·6, p=0·28) lower in 2014 than in 2012.

Mean vaccine coverage was 26% in infants who tested negative for rotavirus Jan 1 to June 30, 2013, rising to 92% for the equivalent period in 2014. In January to June, 2012, incidence of rotavirus hospital admission per 100 000 infants was 269, whereas it was 284 in the same months of 2013 (rise of 5·8%, 95% CI −23·1 to 45·4; p=0·73) and 153 in these months in 2014 (a reduction from the prevaccine period of 43·2%, 18·0–60·7; p=0·003; appendix). Poisson regression of incidence of rotavirus hospital admission in infants against population vaccine coverage for the entire surveillance period Jan 1, 2012, to June 30, 2014, and adjusted for calendar year and month, showed a reduction in incidence of 6·6% (3·7–9·5; p<0·0001) for a 10% increase in vaccination coverage. Incidence in children aged 1–4 years fell by 10·9% (−7·7 to 29·1; p=0·25; appendix).

For the case-control portion of our study, we recruited 118 vaccine-eligible children with rotavirus gastroenteritis (median age 8·9 months, IQR 6·1–11·1, range 2·5–17·0), 317 rotavirus-test-negative children with gastroenteritis (9·4 months, 6·9–11·9, 2·3–18·0), and 380 community controls (8·8 months, 6·5–11·1, 1·4–18·2; table 1). Sociodemographic characteristics of recruited children did not differ between cases and controls (table 1). 109 children (93%) with rotavirus had a vaccine record, as did 283 (90%) of rotavirus-test-negative controls and 370 (97%) community controls. Age of rotavirus vaccine receipt did not differ between children with rotavirus and those without (table 1). 87% of infants received dose one by 10 weeks and 98% within the nationally mandated limit of 15 weeks; 78% received dose two by 14 weeks and none received dose two beyond 26 weeks (appendix). Vaccine coverage did not significantly differ between children with rotavirus and either control group for any antigen except for rotavirus (table 1).

**Table 1:** Demographic and clinical characteristics of children

			**Rotavirus-positive children (n=118)**	**Negative controls (n=317)**		**Community controls (n=380)**	
				Rotavirus-negative children	p for difference	Community controls	p for difference
Median age (months)			8·9 (6·1–11·1)	9·4 (6·9–11·9)	0·16*	8·8 (6·5–11·1)	0·81*
Median household size			4 (3–6)	5 (4–6)	0·31*	4 (3–5)	0·44
Median maternal age			26 (22–30)	25 (22–30)	0·70*	27 (21–30)	0·19*
Maternal orphan			4 (3%)	7 (2%)	0·50	0	0·01†
Paternal orphan			1 (1%)	3 (1%)	0·93	1 (0·3%)	0·40†
Preterm birth (<37 weeks)			5 (4%)	15 (5%)	0·81	··	··
Median birthweight (kg)			3·0 (2·8–3·5)	3·0 (2·7–3·3)	0·09	··	··
HIV exposed‡			20 (17%)	66 (21%)	0·44	··	··
HIV infected			5 (4%)	14 (5%)	0·92	··	··
Currently breastfed			107 (91%)	286 (92%)	0·39	··	··
Exclusively breastfed			5 (4%)	16 (6%)	0·71	··	··
Mean weight for age *Z* score§			−1·3 (1·5)	−1·7 (1·6)	0·05	−0·2 (1·2)	<0·0001
Mean length for age *Z* score			0·4 (2·7)	0·2 (2·9)	0·60	−1·8 (1·9)	<0·0001
Diarrhoea			118 (100%)	317 (100%)	··	0	··
Admitted inpatient			104 (89%)	286 (91%)	0·55	0	··
Vesikari score					0·003¶		
	≤10		25 (21%)	11 (35%)	··	··	··
	10–14		57 (49%)	139 (44%)	··	··	··
	≥15		35 (30%)	64 (20%)	··	··	··
Mean Vesikari score			12·3 (3·4)	11·3 (3·5)	0·01	··	··
Verified vaccination status			109 (93%)	283 (90%)	0·30	370 (97%)	0·03
Vaccine coverage among children with verified vaccination status							
	Rotavirus vaccine				0·03‡		0·04¶
		0 doses	16 (15%)	17 (6%)	··	28 (8%)	··
		1 dose	9 (8%)	30 (11%)	··	42 (12%)	··
		2 doses	81 (75%)	234 (83%)	··	286 (80%)	··
		Missing data	2	1	··	2	··
		Median age at dose 2 (weeks)	12·4(11·1–15·3)	12·0(10·9–15·9)	0·46*	12·8 (11·0–14·6)	0·45*
	Pentavalent vaccine				0·82‡		0·63‡
		0 doses	2 (2%)	4 (1%)	··	6 (2%)	··
		1 dose	6 (6%)	14 (5%)	··	19 (5%)	··
		2 doses	6 (6%)	26 (9%)	··	43 (12%)	··
		3 doses	94 (87%)	235 (83%)	··	288 (78%)	··
		Missing data	0	3	··	14	··
	Oral polio vaccine (birth dose)		83 (77%)	207 (73%)	0·49	283 (76%)	0·14
	Oral polio vaccine				0·85¶		0·61‡
		0 doses	2 (2%)	4 (1%)	··	10 (3%)	··
		1 dose	7 (6%)	16 (6%)	··	27 (7%)	··
		2 doses	15 (14%)	42 (15%)	··	50 (14%)	··
		3 doses	84 (78%)	219 (78%)	··	269 (73%)	··
		Missing data	0	1	··	14	··
	Pneumococcal vaccine				0·64¶		0·06‡
		0 doses	0	3 (1%)	··	7 (2%)	··
		1 dose	4 (4%)	7 (3%)	··	22 (6%)	··
		2 doses	8 (7%)	28 (10%)	··	41 (11%)	··
		3 doses	91 (84%)	233 (83%)	··	286 (77%)	··
		Missing data	5	11	··	14	··
	BCG vaccine		104 (96%)	272 (96%)	0·80	346 (94%)	0·18
	Measles vaccine		32 (29%)	96 (34%)	0·37	113 (31%)	0·88

Data are median (IQR), n (%), or mean (SD), unless otherwise shown.

* Wilcoxon rank-sum test.

† Fisher's exact test.

‡ HIV exposed is defined in appendix.

§ Anthropometry at presentation to hospital or at recruitment in the community.

¶ Mantel-Haenszel χ^2^ test.

Vaccine effectiveness for two doses of RV1 was 64% (95% CI 24–83) in test-negative control individuals and 63% (23–83) in community controls (table 2). For children with more severe disease (Vesikari score ≥11), effectiveness for two doses of RV1 was 68% (95% CI 22–87) in test-negative control individuals and 68% (23–86) in community controls (table 2). The two-dose vaccine effectiveness point estimate was higher for rotavirus G1 (82% [42–95] and 78% [8–95] for test-negative and community controls, respectively), than for rotavirus G2 (53% [–28 to 83] and 61% [–29 to 88], respectively), or for rotavirus G12 (53% [–99 to 89] and 61% [–208 to 95], respectively; table 2).

**Table 2:** Rotavirus vaccine effectiveness by dose of rotavirus vaccine

		**Rotavirus-positive children**	**Rotavirus test-negative controls**		**Community controls**	
			Patients	Adjusted vaccine effectiveness*	Patients	Vaccine effectiveness†
**Children eligible for dose 2**						
Number for assessment		109	283	··	356	··
Median age (months)		8·2 (6·6–10·9)	9·0 (6·6–11·6)	··	8·2 (6·5–11·0)	··
Number of doses						
	0 doses	16 (15%)	17 (6%)	Ref	28 (8%)	Ref
	2 doses	81 (74%)	234 (83%)	64% (24–83)	286 (80%)	63% (23–83)
	>1 dose	90 (83%)	264 (94%)	65% (27–83)	328 (90%)	68% (42–83)
**Children with Vesikari score ≥11**						
Number for assessment		90	197	··	288	··
Median age (months)		8·6 (6·6–11·0)	9·5 (7·3–11·4)	··	8·6 (6·5–11·0)	··
Number of doses						
	0 doses	13 (14%)	10 (5%)	Ref	19 (7%)	Ref
	2 doses	69 (77%)	195 (89%)	68% (22–87)	239 (83%)	68% (23–86)
	>1 dose	77 (89%)	208 (95%)	69% (25–87)	269 (91%)	68% (37–83)
**Non-mixed G1 rotavirus infection**						
Number for assessment		21	283	··	66	··
Number of doses						
	0 doses	5 (24%)	18 (6%)	Ref	6 (9%)	Ref
	2 doses	12 (57%)	234 (83%)	82% (42–95)	53 (80%)	78% (8–95)
**Non-mixed G2 rotavirus infection**						
Number for assessment		50	283	··	167	··
Number of doses						
	0 doses	6 (12%)	18 (6%)	Ref	11 (7%)	Ref
	2 doses	38 (76%)	234 (83%)	53% (−28 to 83)	135 (81%)	61% (−29 to 88)
**Non-mixed G12 rotavirus infection**						
Number for assessment		18	283	··	48	··
Number of doses						
	0 doses	3 (17%)	18 (6%)	Ref	5 (10%)	Ref
	2 doses	14 (78%)	234 (83%)	53% (−99 to 89)	34 (71%)	61% (−208 to 95)

Data are n (%), median (IQR), and vaccine effectiveness (95% CI).

* Adjusted for age at admission and month and year of birth.

† Restricted to controls that were matched within 30 days before or after the case's date of birth if they were younger than 1 year and 3 months if they were older than 1 year.

## Discussion

We show that RV1 reduces the number of hospital admissions for acute rotavirus gastroenteritis in Malawi, one of the first African countries with high rotavirus-associated mortality to implement routine infant vaccination for rotavirus. We detected vaccine effectiveness that was at least equal to that reported in the RV1 efficacy trial in Malawi.^10^ In the first year after vaccine introduction, a sharp rise occurred in hospital admissions for rotavirus in children younger than 5 years, but there was no significant increase in infants, implying vaccine effect at low vaccine coverage. By 2014, vaccine coverage in infants was high, and the peak incidence during that period was lower than in previous periods. Adjusting for month, we also show an independent inverse dose–response relation between increasing population vaccine coverage and incidence of rotavirus hospital admission in infants; this was not present in older children in whom population coverage occurred later and was much lower. The sustained and more pronounced reduction in rotavirus hospital admission rate and detection rate in the second year after vaccine introduction in 2014, largest in infants, together with a shift in age distribution of rotavirus cases by the second year after vaccine introduction when vaccine coverage was high, suggests early vaccine impact.

Our finding of effectiveness with a 6 and 10 week of age schedule of vaccination, with each dose given in a timely fashion during programmatic roll-out (appendix), strongly supports use of this schedule as recommended by WHO.^13^ The degree of protection afforded by this schedule had not been established previously because RV1 doses were given at 10 and 14 weeks of age in the African efficacy trial and at 6 and 14 weeks in an effectiveness study in South Africa (panel).^10^, ^24^ Indeed in a 2010 study in South Africa, the immunogenicity of two RV1 doses given at 6 and 10 weeks of age was lower than that of two doses given at 10 and 14 weeks (seroconversion 36% *vs* 60%, respectively).^25^ Confirmation of vaccine effectiveness with schedule at 6 and 10 weeks of age is especially important in infants in low-income countries such as Malawi, in which three-quarters of the total rotavirus disease burden is sustained in the first year of life and early protection is needed.^26^, ^27^

Panel **Research in context**

**Systematic review**

We searched Medline using the following query “rotavirus vaccin* AND (efficacy OR effectiveness OR impact) NOT cost-effectiveness” restricted to studies in children published in English in the past 10 years. A randomised controlled trial reported efficacy for the monovalent rotavirus vaccine against severe acute rotavirus gastroenteritis in Malawi. Post-introduction population effectiveness has been shown in high-income and middle-income countries in Europe; North, South, and Central America; Australia; and recently in South Africa. However, no data have been reported from low-income countries in sub-Saharan Africa where there are high burdens of rotavirus gastroenteritis and mortality. Furthermore, no study has shown the effectiveness of rotavirus vaccine given at the WHO-recommended accelerated schedule of 6 and 10 weeks of age.

**Interpretation**

Data are needed to establish definitively the WHO-recommended schedule in high-burden low-income sub-Saharan African countries that are eligible for Gavi-supported vaccine introduction. We present the first such evidence for the effect of the monovalent rotavirus vaccine on rotavirus-associated hospital admissions and present results for a broad range of rotavirus genotypes. Our findings show that in the context of high vaccine coverage in Malawi, the promising data from vaccine efficacy trials have translated into programmatic reductions in rotavirus hospitalisations in a high-disease burden infant population. These data strongly support the continued roll-out of rotavirus vaccines in other low-income countries in Africa and Asia.

We show robust effectiveness of the G1P[8] RV1 vaccine despite genotypic rotavirus diversity in the population, with fully homotypic (G1P[8]), fully heterotypic (G2P[4], G2P[6], and G12P[6]), and partially heterotypic (G12P[8]) genotypes circulating during the study. However, the point estimate for vaccine effectiveness was higher against G1 than against G12 or G2 genotypes. In the second year after vaccine introduction the number of cases of gastroenteritis associated with rotavirus genotypes G1 and G12 decreased, but infections with G2 persisted. Although our study was not powered to assess strain-specific differences in vaccine effectiveness, these data underscore the need for further surveillance to address the potential for lower vaccine effectiveness against fully heterotypic genotypes including G2P[4],^28^, ^29^ particularly since previous findings suggested that RV1 leads to cross-genotype protection in this and other populations.^30^

The point estimate of vaccine effectiveness (64% for hospital-test-negative controls and 63% for community controls) is at the upper limit of that reported by investigators of a randomised trial of RV1 (vaccine effectiveness 49%, 95% CI 19–68) in the same setting in Malawi. Similar magnitudes of vaccine effectiveness were established by comparisons with hospital and community controls. Notably, our study was done in the 2 years immediately after vaccine introduction and thus our population was enriched with young infants in whom vaccine effectiveness might be greatest; further surveillance of children after 2 years of age will be important since protection has been postulated to wane in the second year in low-income settings, although recent studies in South Africa showed sustained protection in the second year of life.^24^, ^31^

Our study has limitations. First, although we show a dose–response relation between population vaccine coverage and reductions in disease incidence that is independent of time, the incidence analysis was based on population denominator projections from census data that are 4 and 14 years old and the numerator assumes no change in health-seeking behaviour. Although both measurements will include error, the nature of the error should be consistent over time and not affected by RV1 introduction. Nonetheless these data should be interpreted cautiously, based as they are on a short period of observation before vaccine introduction and the observation of low incidence during the prevaccine period in older children. Second, our case-control analysis examined only the direct protection provided by vaccination. The total benefit of a national rotavirus vaccine programme is probably greater than that provided by direct protection, as has been noted in other settings after rotavirus vaccine introduction.^32^, ^33^ Blantyre is an urban site with periurban rural areas. Our results might not be representative of sociodemographically or economically differing settings.
