# Supplementary material for: Effectiveness of a monovalent rotavirus vaccine in infants in Malawi after programmatic roll-out: an observational and case-control study
Source: Lancet Infect Dis. 2015 Apr;15(4):422–8. doi: 10.1016/S1473-3099(14)71060-6 (PMC4374102; doi:10.1016/S1473-3099(14)71060-6)

THE LANCET Infectious Diseases

Supplementary webappendix

This webappendix formed part of the original submission and has been peer reviewed. We post it as supplied by the authors.

Supplement to: Bar-Zeev N, Kapanda L, Tate JE, et al. Effectiveness of a monovalent rotavirus vaccine in infants in Malawi after programmatic roll-out: an observational and case-control study. *Lancet Infect Dis* 2015; published online Jan 29. http://dx.doi.org/10.1016/S1473-3099(14)71060-6.

Web Extra Table 1. Definitions

Gastroenteritis/Diarrhoea	Passage of stools of a less formed character than usual for that child occurring in 3 or more episodes within 24 hours.
Rotavirus confirmed case	Vaccine age-eligible child presenting to QECH with diarrhoea and stool has positive rotavirus result on ELISA.
Test-negative	Vaccine age-eligible child presenting to QECH with diarrhoea and stool has negative rotavirus result on ELISA.
Hospital admission	Children staying overnight in hospital, or children requiring observation of >4 hours in hospital short stay unit.
Vaccine age-eligible	Born on or after 17 September 2012 – age less than 6 weeks on date of vaccine introduction on 29 October 2012.
HIV-exposed	Any child born to an HIV-infected mother, or an infant <12 months old with a positive antibody test and absent HIV DNA PCR result or negative result in any infant still breastfeeding.
HIV-infected	Infant <12 months with positive HIV DNA PCR, or child 12 months or older with two sequential positive rapid antibody tests.
Dehydration (for Vesikari score)	None: Absence of any clinical sign of dehydration on admission.<br>Mild-Moderate: Presence on admission of two or more of:<br>• Restless or irritable<br>• Sunken eyes<br>• Thirsty or drinking eagerly<br>• Slow skin pinch<br>Severe: Presence on admission of two or more of:<br>• Lethargic or unconscious<br>• Sunken eyes<br>• Drinks poorly or is unable to drink<br>• Very slow skin pinch

QECH = Queen Elizabeth Central Hospital
ELISA = Enzyme-linked immunosorbent assay
HIV DNA PCR = Human Immunodeficiency Virus deoxyribonucleic acid polymerase chain reaction

Web Extra Table 2. Inclusion and exclusion criteria

Inclusion criteria	Case-patients were eligible for enrolment in the surveillance program if they met ***all*** of the following criteria:<br>1. Are <5 years of age.<br>2. Live in Blantyre district.<br>3. Present to QECH with diarrhoea on or after the study start date<br>4. Clinical illness should not be explained by an alternative underlying medical condition.<br>5. Clinical illness commenced within 14 days prior to hospital visit; and<br>6. Are either:<br>a. Seen at QECH and treated with rehydration (oral or intravenous) for diarrhoea and discharged home; or<br>b. Admitted to the hospital and treated for diarrhoea.<br>7. Eligible to have received at least one dose of vaccine prior to illness (i.e. age 8 weeks or older and following date of vaccine introduction).<br>8. Produce a rotavirus positive stool sample during the first 48 hours after presentation.
Exclusion criteria	Case-patients were excluded from enrolment in the active surveillance program if ***any*** of the following applied:<br>1. Unable to contact parent or guardian to obtain informed consent.<br>2. Admitted to another hospital for >24 hours (and subsequently transferred to QECH).<br>3. Re-presentation within 14 days of previous hospital separation for the same illness.<br>4. Hospital admission >48 hours prior to enrolment.<br>5. Known oncological diagnosis or congenital immunodeficiency (apart from HIV infection).<br>6. Enrolled prior to vaccine introduction.<br>7. Age-ineligible to have received vaccine.

QECH = Queen Elizabeth Central Hospital

Web Extra Table 3. Demographic and clinical characteristics of children younger than 5 years presenting to Queen Elizabeth Central Hospital, Blantyre Malawi by case status, enrolled 1 January 2012 – 30 June 2014

	**Rotavirus Positive N=508 (%)**	**Rotavirus Negative N=933 (%)**	***P*-value**
Median age in months (range)	10•0 (0•5–41•9)	10•8 (0•4–58•9)	<0•001[a]
Median household size (range)	4 (2–12)	4 (2–15)	0•22[a]
Median maternal age (range)	25 (15–54)	25 (16–42)	0•64[a]
Maternal orphan	5 (1%)	16 (2%)	0•27
Paternal orphan	5 (1%)	15 (2%)	0•34
Preterm birth (<37 weeks)	13 (2.6%)	46 (5%)	0•81
Median birth weight (kg) (range)	3 (1•5–5•5)	3 (1•1–5•0)	0•03
HIV exposed	82 (17%)	175 (19%)	0•44
HIV infected	20 (5%)	59 (8%)	0•05
Currently breast fed	456 (91%)	729 (79%)	<0•001*
Exclusively breast fed	38 (8%)	45 (6%)	0•17
Mean weight for age Z-score (SD) †	-1•2 (1•3)	-1•6 (1•6)	<0•001
Mean length for age Z-score (SD)	-0•7 (2•6)	-0•6(3•1)	0•66
Diarrhoea	508 (100%)	933 (100%)	
Admitted inpatient	465 (92%)	844 (90%)	0•50
Vesikari Score			<0•001[b]
≤10	98 (19%)	317 (34%)	
10-14	301 (59%)	461 (47%)	
≥15	109 (21%)	153 (16%)	
Mean (SD)	12•0 (3•4)	11•2 (3•4)	<0•001
Verified Vaccination Status	459 (91%)	795 (85%)	0•006*

a Wilcoxon Rank Sum test, **b** Mantel-Haenszel χ^2 test, SD= Standard Deviation
* Association lost once adjusted for age
† Anthropometry at presentation to hospital

Web Extra Figure 1. Recruitment flow diagram of children presenting with diarrhoea to Queen Elizabeth Central Hospital, 1 January 2012 to 30 June 2014.

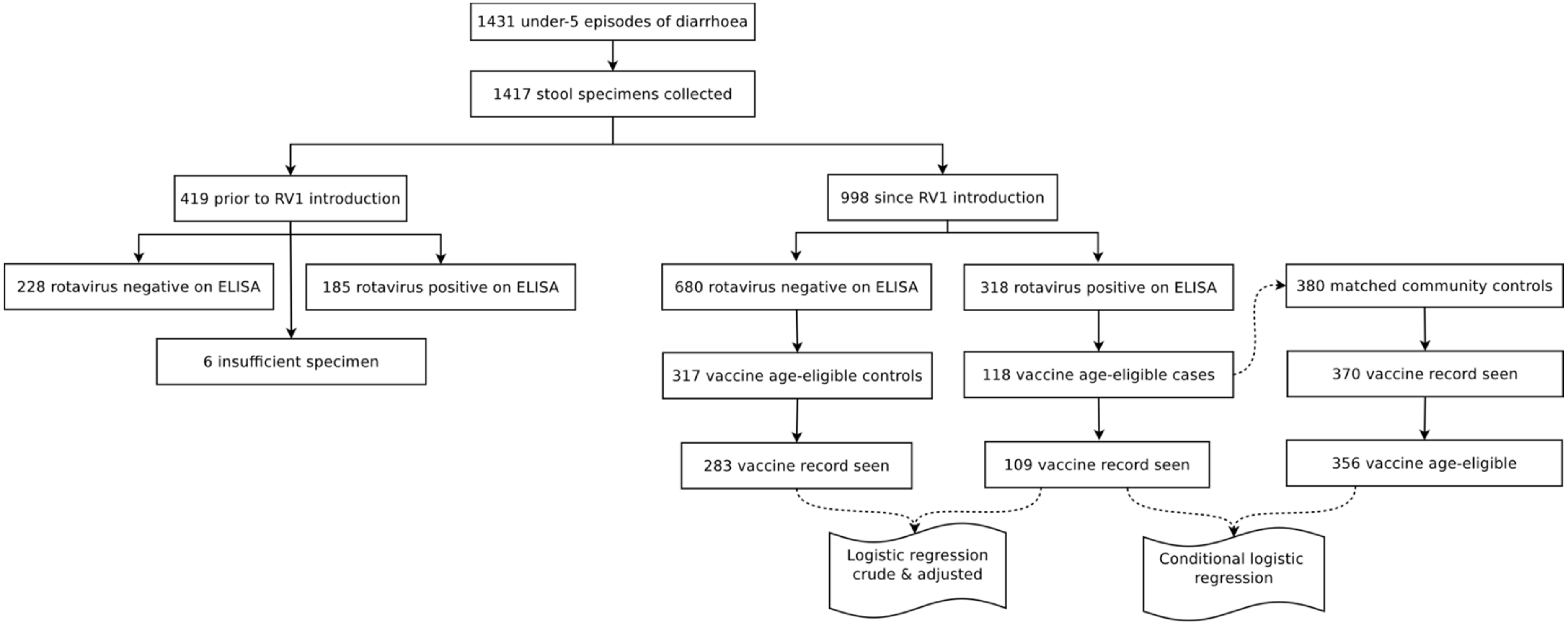

Web Extra Figure 2. Age distribution of children <5years with rotavirus confirmed diarrhoea before vaccine introduction (1 January 2012 to 28 October 2012) and after vaccine introduction (29 October 2012 to 30 June 2014).

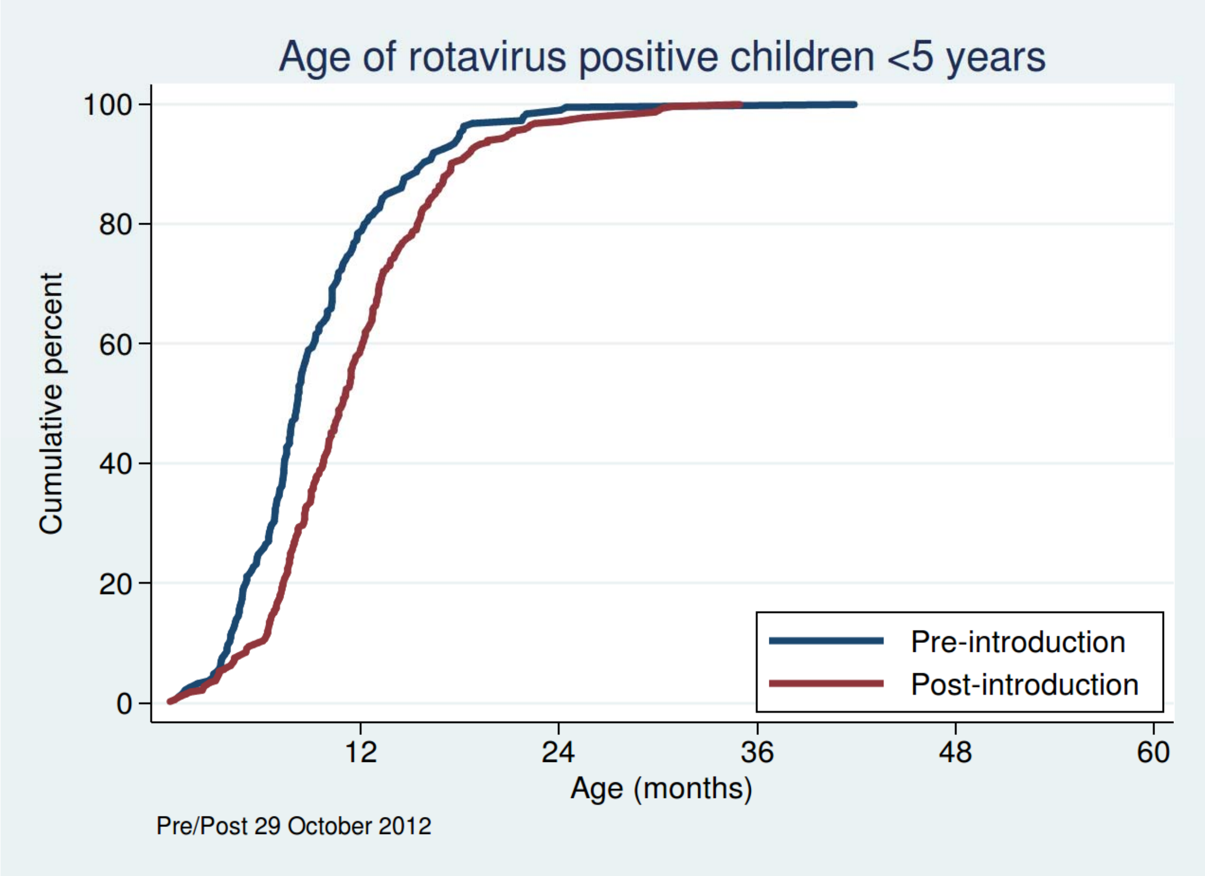

Web Extra Figure 3. Rotavirus genotypes among children <5 years of age presenting to Queen Elizabeth Central Hospital, Blantyre. Includes pre-rotavirus vaccine period (1 January 2012 to 28 October 2012) and post-rotavirus vaccine period (29 October 2012 to 30 June 2014).

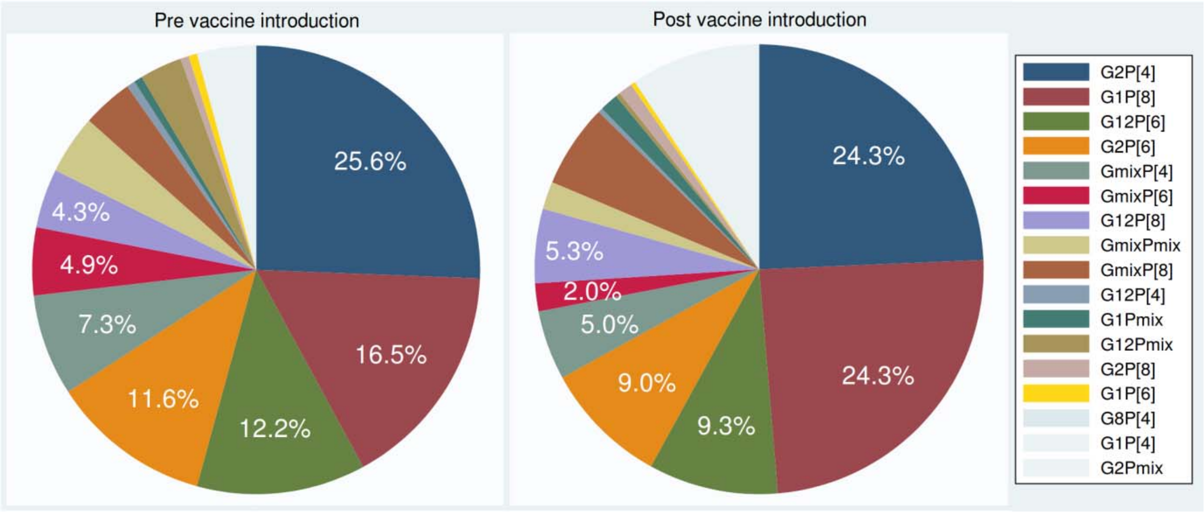

Web Extra Figure 4. Rotavirus hospitalization incidence and vaccination population coverage among infants and children, 1 January 2012 to 30 June 2014.

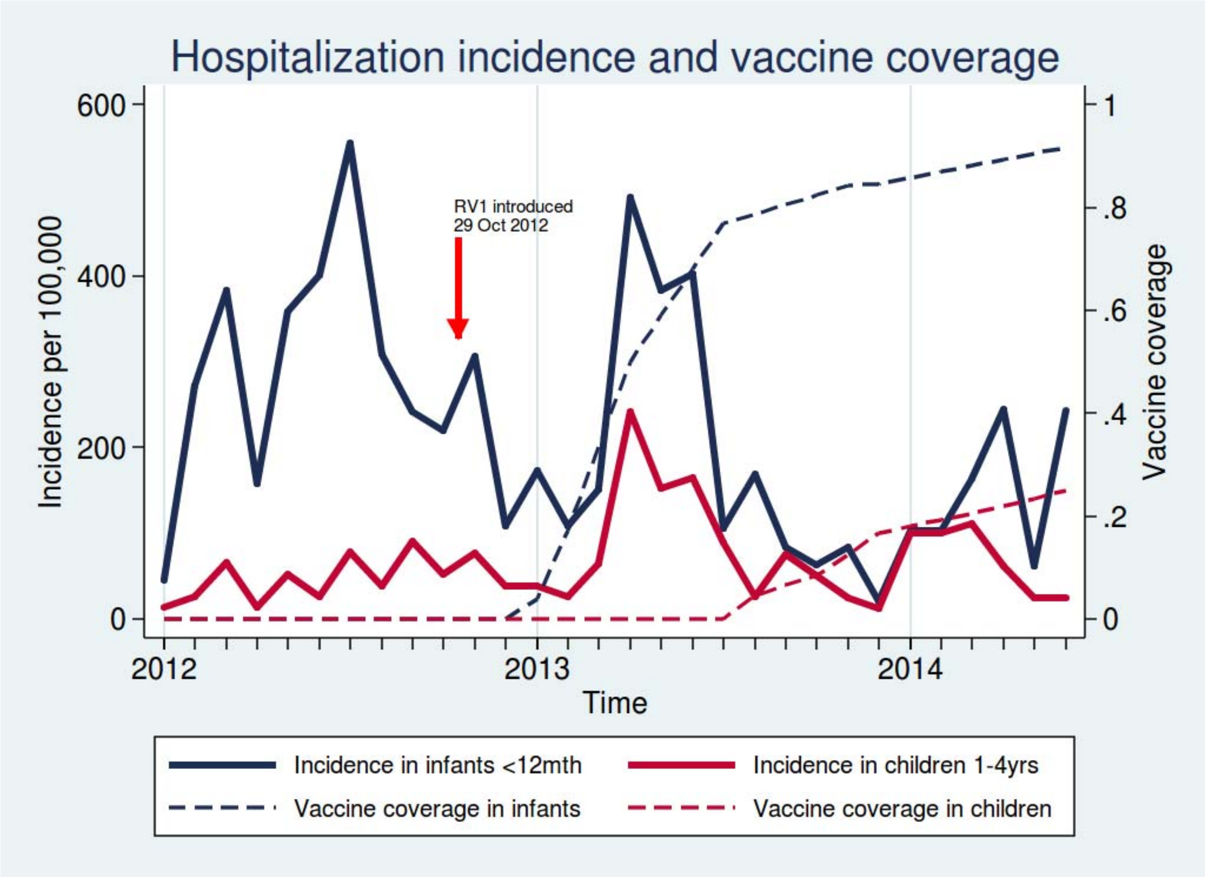

Web Extra Figure 5. Age of rotavirus vaccination among recruited children presenting with diarrhoea to Queen Elizabeth Central Hospital, 1 January 2012 to 30 June 2014. DTP-HBV-HiB vaccine is shown for comparison.

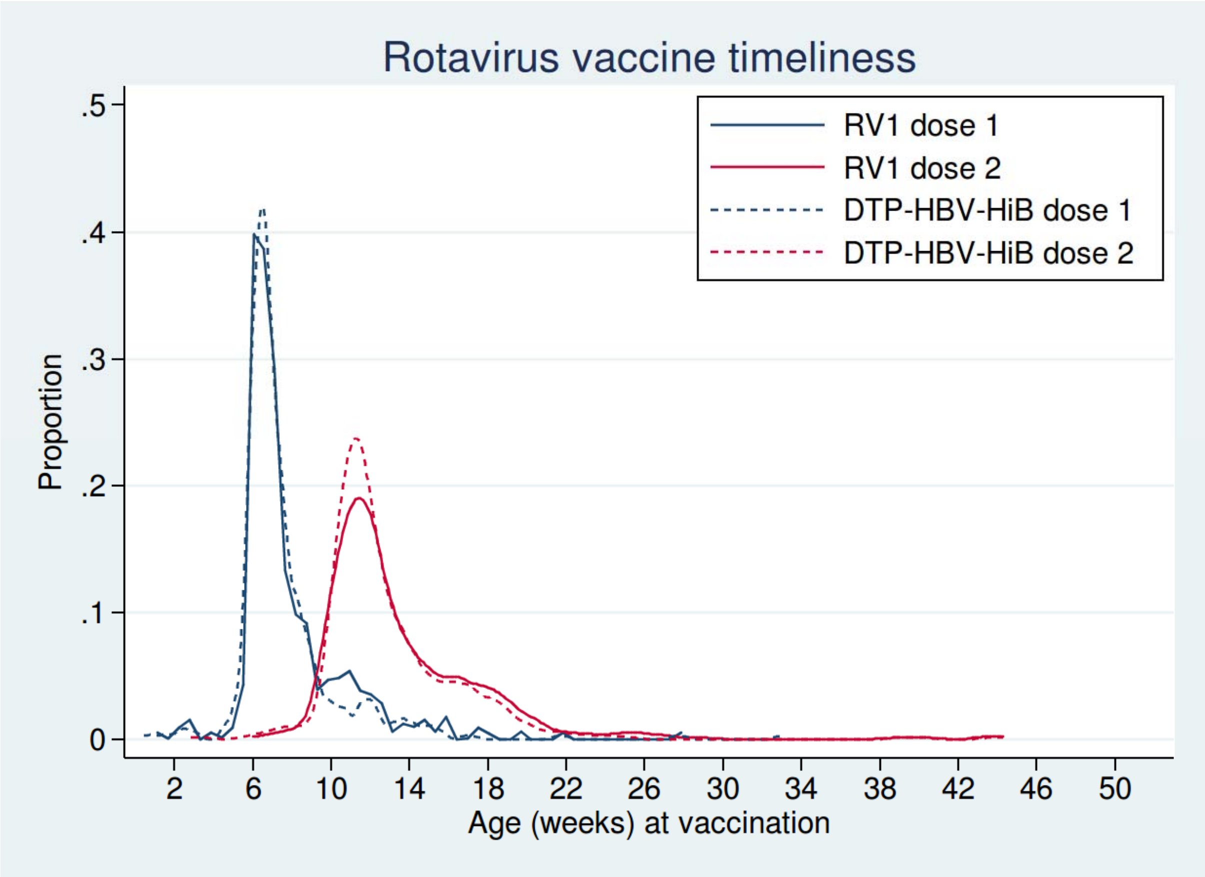

Supplement: Supplementary appendix [file mmc1.pdf]
